# Three Cases of Levodopa–Carbidopa Responsive Nocturnal Akinesis in Male Children of an Inbred Family in Rural Baluchistan, Pakistan

**DOI:** 10.3390/ijms27209068

**Published:** 2026-10-13

**Authors:** Javed Akram, Amama Ghaffar, Muhammad A. Usmani, Usman Shareef, Zubair M. Ahmed, Sheikh Riazuddin, Saima Riazuddin

**Affiliations:** 1 Pakistan Society of Internal Medicine, Justice Akram Road, Lahore 54600, Pakistan; 2 Jinnah Burn and Reconstructive Surgery Center, Allama Iqbal Medical College, University of Health Sciences, Lahore 54550, Pakistan; 3 Department of Otorhinolaryngology Head & Neck Surgery, School of Medicine, University of Maryland, Baltimore, MD 21201, USA; zmahmed@som.umaryland.edu; 4 Department of Molecular Biology, Shaheed Zulfiqar Ali Bhutto Medical University, Islamabad 44000, Pakistan; 5 Shifa College of Pharmaceutical Sciences, Shifa Tameer-e-Millat University, Islamabad 44000, Pakistan; 6 Department of Biochemistry and Molecular Biology, School of Medicine, University of Maryland, Baltimore, MD 21201, USA; 7 Marlene & Stewart Greenebaum Comprehensive Cancer Center, School of Medicine, University of Maryland, Baltimore, MD 21201, USA

**Keywords:** nocturnal akinesis, tyrosine hydroxylase deficiency, dopa-responsive dystonia, solar kids

## Abstract

After more than 9 years of follow-up, we describe three affected male siblings from a consanguineous family (PKSOK) in rural Baluchistan, Pakistan, who presented with levodopa–carbidopa responsive nocturnal akinesia associated with tyrosine hydroxylase deficiency. The affected individuals also show delayed motor milestones, mild intellectual disability, speech and psychomotor delay, without epilepsy, spasticity, cerebellar ataxia, or daytime motor weakness. The marked improvement in nocturnal motor symptoms following levodopa–carbidopa (100 mg/25 mg) treatment raised the possibility of an underlying disorder of dopamine biosynthesis. Exome sequencing of the two affected siblings (IV:16 and IV:18) revealed twenty rare (frequency < 0.001) homozygous coding variants shared between them. However, segregation analysis with the disease phenotype ruled out the potential involvement of all these twenty variants. Therefore, we extended the analysis beyond the coding region variants to non-coding variants, which revealed a homozygous variant, c.-70G>A, in the regulatory region of *TH* (tyrosine hydroxylase). The c.-70G>A variant has been previously reported in three studies; however, our study represents the first report in the South Asian population. Our study also expands the phenotypic spectrum of disorders associated with variants in *TH* and highlights the importance of considering variants in regulatory regions of *TH* in patients with atypical but levodopa-responsive neurological phenotypes.

## 1. Introduction

In 2015, a family from a remote rural area of Baluchistan, Pakistan, was reported in the local media because of an unusual neurological phenotype affecting two siblings. The affected individuals were described as having a marked loss of voluntary motor function during the night, while remaining comparatively functional during the daytime. At that time, a third sibling, younger than one year of age, had not yet been diagnosed with the same condition. Due to the remarkable day–night variation in their symptoms, the siblings were later widely referred to as the “Solar Kids” in the media [1].

Rare familial disorders with atypical clinical presentations remain difficult to diagnose, particularly when the phenotype does not fully correspond to relatively rare Mendelian conditions. Although exome sequencing has improved the molecular diagnosis of rare diseases, a proportion of clinically distinctive familial phenotypes remains unresolved because of limitations in detecting non-coding, regulatory, structural, mitochondrial, and epigenetic disease mechanisms [2,3,4]. Following institutional review board approval, we conducted longitudinal clinical and molecular investigations of this family, with follow-up extending over more than nine years. The affected individuals showed substantial clinical improvement following initiation of levodopa–carbidopa therapy, which helped normalize their nocturnal akinesia. This suggested an underlying dopamine biosynthesis defect, which led to the identification of the known regulatory *TH* variant c.-70G>A (GRCh37/hg19, NM_000360.4, chr11:2193086C>T). Hence, the combination of clinical care and comprehensive genetic screening provided the precise diagnosis of the phenotype segregating in family PKSOK.

## 2. Detailed Case Description

### 2.1. Clinical Presentation and Follow-Up

**Figure 1:**
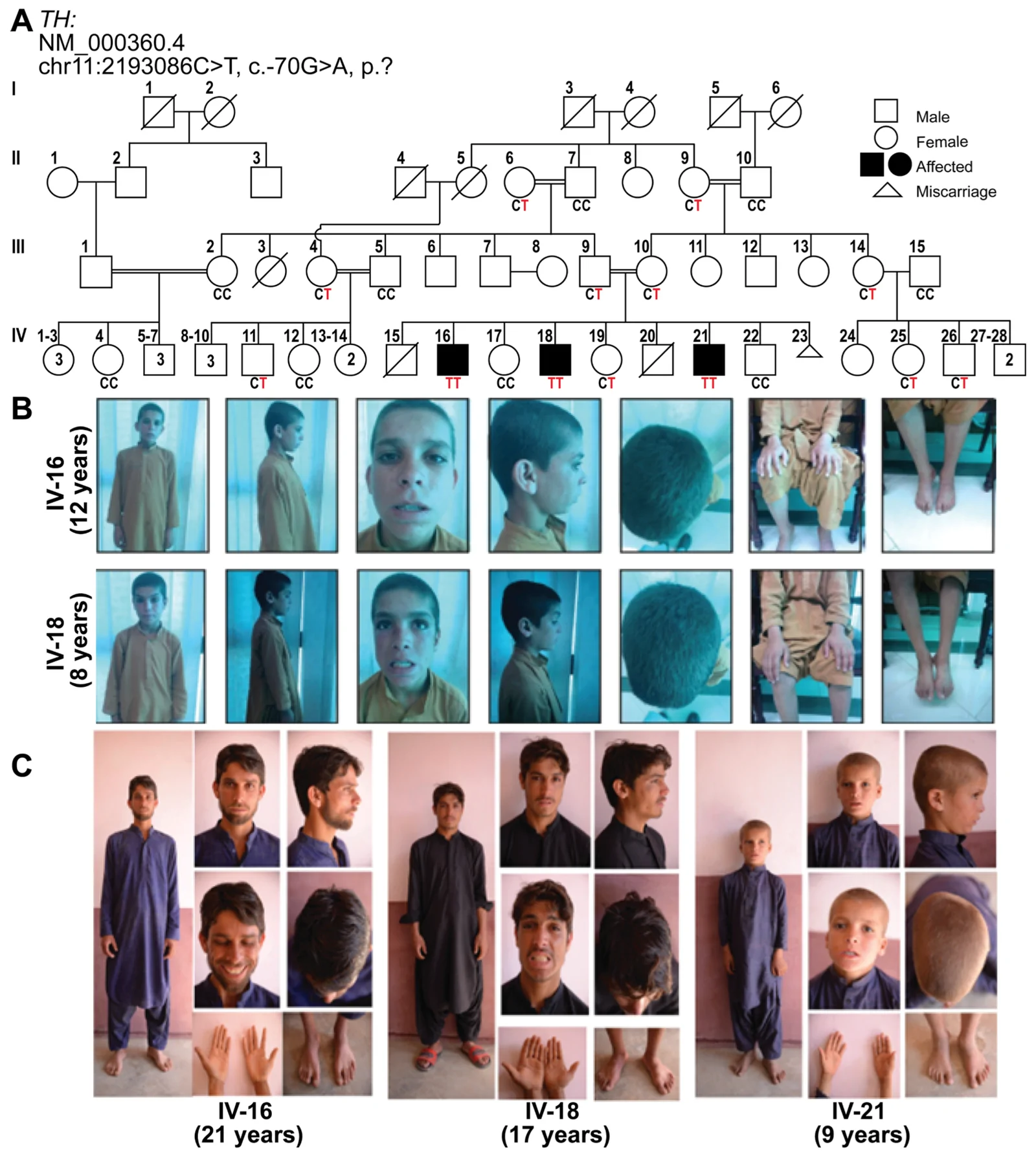
Pedigree and photographs of family PKSOK. (**A**) Family structure of PKSOK showing segregation for *TH* variant c.-70G>A. Double horizontal lines represent consanguineous marriages; affected individuals are shown by filled symbols. Males are represented as squares and females as circles. A crossed line over symbols denotes a deceased individual, and the bridge/crossover line indicates that one pedigree line passes over another without genealogical intersection at the crossing point. (**B**) Pictures of two affected male siblings at their first diagnosis in 2016. (**C**) Pictures of two affected male siblings along with their 3rd sibling during a recent follow-up visit.

Three male siblings from a consanguineous Pakistani family, aged 9, 17, and 21 years at the most recent assessment, had childhood-onset nocturnal akinesia. This nocturnal manifestation was characterized by markedly reduced voluntary motor activity, accompanied by generalized muscle weakness and hypotonia, rather than isolated akinesia (Figure 1; Table 1).

Examination of the cranial nerves revealed partial loss of motor function. The affected individuals remained well-oriented and exhibited normal sensory function and tendon reflexes during both nocturnal episodes and daytime examinations. Muscle strength, voluntary movements and muscle tone improved during the day or after the administration of oral levodopa–carbidopa (100 mg/25 mg) at night, as detailed in Table 1.

Neurological assessments were documented before and after treatment. Improvement in motor function was observed following treatment, with no adverse effects noted (Table 1).

For all three individuals, the condition started during the first year of life, characterized by a state of lethargy in the evening. The affected individuals could not walk until after 2 years of age. During the nocturnal episodes, their speech would get slow and their axial muscles showed pronounced weakness, due to which they were unable to sit and hold their heads upright (Table 1).

**Figure 2:**
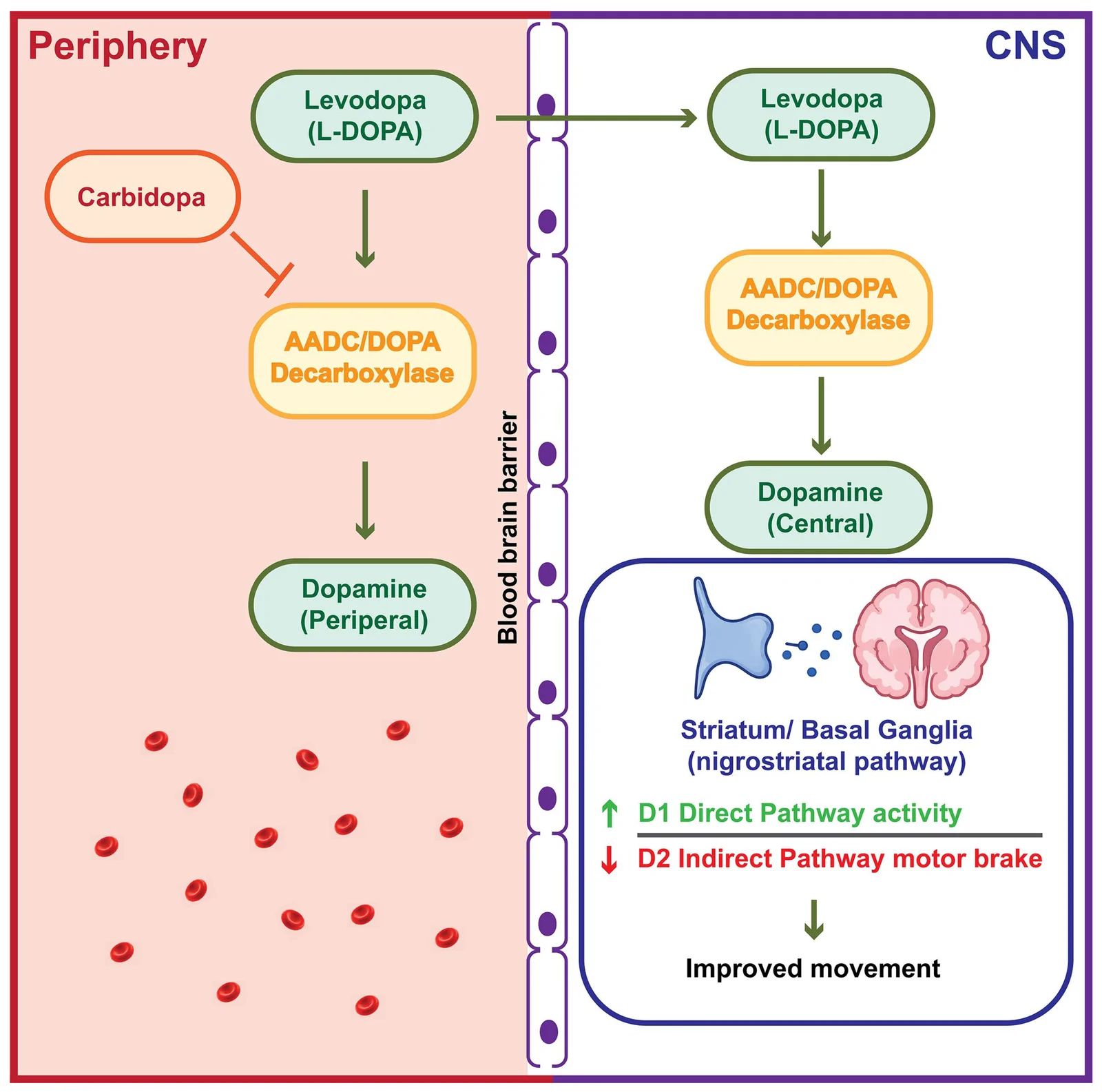
The general mechanism of action and pharmacological effects of levodopa–carbidopa. Carbidopa inhibits peripheral aromatic L-amino acid decarboxylase (AADC/DOPA decarboxylase), reducing the peripheral conversion of levodopa to dopamine. This increases the availability of levodopa to cross the blood–brain barrier and enter the central nervous system (CNS). In the CNS, levodopa is converted to dopamine by AADC/DOPA decarboxylase, increasing central dopamine availability. Dopamine acts within the striatum and basal ganglia nigrostriatal pathway to enhance D1 receptor-mediated direct pathway activity and reduce D2 receptor-mediated indirect pathway motor inhibition, thereby facilitating movement.

Clinical history and assessment of the three siblings suggested mild intellectual disability based on everyday adaptive functioning and longitudinal clinical observations; however, formal standardized psychometric assessments were not performed. The affected individuals remained well-oriented during nocturnal episodes, although this did not necessarily reflect normal overall intellectual functioning. There was no history of diplopia, difficulty in swallowing, involuntary movements or birth asphyxia. At night, the siblings were dependent on their caretakers for feeding and defecation. There was no history of seizures, ataxia and fecal or urinary incontinence. The weakness had progressively worsened over the years, and the eldest one was the most affected. There was no evidence of ptosis, extraocular ophthalmoplegia or palatal paralysis. The children were afebrile; however, their extremities were warm and sweaty. General physical examination was normal, and no apparent abnormalities of other organs were noted. There was no evidence of respiratory distress, and the abdominal and cardiovascular examinations were unremarkable. A pre- and post-exercise electromyography (EMG) conducted in the morning was unremarkable and showed no decremental response on repetitive nerve stimulation. Other investigations, including complete blood count (Supplementary Table S1), serum biochemistry, nerve conduction studies (NCS), computed tomography (CT), and magnetic resonance imaging (MRI), were unremarkable. Anticholinesterase and antinuclear(ANA) antibodies tests were negative, while the Tensilon test also did not show any improvement. The differential diagnosis included hereditary dystonia (MIM#128100), myasthenia gravis (MIM#254210), and periodic paralysis (MIM#170400), including hypokalemic periodic paralysis. Intriguingly, the administration of a low dose of levodopa (Figure 2) resulted in improvement of symptoms, and currently, the symptoms are managed through regular use of levodopa.

Based on the pattern of inheritance in the family pedigree (Figure 1), both autosomal recessive and X-linked recessive inherited disorders were considered in the analysis of exome sequencing data from two affected individuals (IV:16 and IV:18). X-linked recessive inheritance was considered because all affected individuals were male, and the underlying genetic diagnosis was unknown at the time of analysis.

Using the criteria of read depth ≥ 4, allele frequency < 0.001, CADD score ≥ 10, and non-synonymous variants, we revealed twenty coding variants shared among the two samples (Supplementary Table S2). All of these variants were Sanger sequenced in the DNA samples of all the participating family members. However, none of these variants co-segregated with the nocturnal akinesia phenotype, which ruled out their candidacy to cause the disease (Supplementary Table S2). We then investigated non-coding variants in genes associated with neurotransmitter disorders, which identified a previously reported homozygous variant, c.-70G>A, in the regulatory region of *TH* (tyrosine hydroxylase), a gene associated with TH deficiency [5,6,7]. However, biochemical testing to detect TH deficiency through CSF L-DOPA, homovanillic acid (HVA) and 5-HIAA could not be performed as the family did not consent to a lumbar puncture. In addition, plasma and urinary testing for catecholamines and their metabolites is not locally available.

### 2.2. Methodology

#### 2.2.1. Family Enrolment & Clinical Investigation

After institutional board review and approval, family PKSOK affected with nocturnal paralysis and mild intellectual disability (ID) was ascertained from the rural area of the Baluchistan province in Pakistan. The clinical diagnosis was made through a series of neurological, morphological, behavioral, ophthalmological, auditory, skeletal, and dermatological assessments. Front and side-pose facial photographs were taken to document clinical and phenotypic features. Peripheral blood samples were collected from the affected individuals, parents, normal siblings, and all available relatives. Written informed consent was obtained from each participant, including the guardians of affected individuals and minors.

#### 2.2.2. Exome Sequencing (ES)

ES was performed at the University of Maryland, Baltimore, USA, as previously described [8]. Briefly, genomic DNA samples from two affected individuals (IV:16 and IV:18) were used to perform exome enrichment and high-throughput parallel sequencing. Exome enrichment was performed using the Nimblegen SeqCap EX Exome v2.0 Library (Roche Diagnostics, San Francisco, CA, USA), followed by generating approximately 100 million paired-end reads with a length of one hundred base pairs. The Illumina HiSeq 4000 platform (Illumina, Inc., San Diego, CA, USA) was used to perform massive parallel sequencing. Raw sequencing data was processed using the Genome Analysis Toolkit (The Broad Institute’s Genome Analysis Toolkit, Broad Institute, Cambridge, MA, USA). Reads passing the Illumina Chastity Filter were aligned to the human reference genome GRCh37/hg19 using the Burrows–Wheeler Aligner (BWA; Broad Institute). GATK was used to call the variant sites, and the variant quality score recalibration method was used to filter the single nucleotide variant (SNV) calls. To prioritize the most relevant candidate variant from ES data, an in-house customized pipeline was used and variants shared by both affected siblings (IV:16 and 18) were selected [9,10]. However, to identify non-coding variants, we used Franklin (https://franklin.genoox.com accessed on 31 August 2026) tool to specifically target genes involved in neurotransmitter disorders using HP:0001939. All these variants were confirmed through Sanger sequencing for segregation among all the affected and unaffected enrolled individuals of the family.

#### 2.2.3. Segregation Analysis

The online primer designing tool Primer3 (Version 4.0) was used to design the primers. For the amplification of identified variants, 2X Econotaq DNA Polymerase (Bioresearch Technologies, Radnor, PA, USA) was used. After PCR amplification of candidate genes, 3 µL of PCR product was loaded on a 2% agarose gel containing EtBr. The size of the amplified bands was determined by running a 1 KB Plus DNA Ladder (Gene Ruler, Thermo Scientific INC., Waltham, MA, USA) in parallel wells. ExoSap was performed followed by Sanger sequencing PCR and precipitation to process and analyze samples using the ABI sequencer. The chromatograms generated were analyzed using SeqMan Pro (DNASTAR Navigator version 18).

## 3. Discussion

The clinical presentation of family PKSOK was marked by pronounced day–night variation in motor function. All three affected individuals developed symptoms during the first year of life, with progressive evening and nighttime akinesia requiring assistance with routine activities, while daytime strength, tone, and movements were relatively preserved. This temporal pattern, together with delayed motor milestones and neurodevelopmental involvement, made the phenotype difficult to classify within a recognized neurological syndrome. Several neuromuscular and movement disorders were initially considered. Myasthenic disorders appeared less likely because of the absence of characteristic ocular, facial, respiratory and bulbar weakness, along with normal electrophysiological studies, and lack of improvement with Tensilon [11,12]. Periodic paralysis was also considered because of the episodic weakness, but the developmental abnormalities, absence of a characteristic potassium-associated paralytic pattern, and subsequent treatment response were not consistent with a typical periodic paralysis syndrome [13,14]. Although childhood-onset dopa-responsive movement disorders were considered, the affected siblings lacked many of the manifestations usually associated with classical dystonia, involving persistent dystonia, spasticity, cogwheel rigidity, gait dystonia, extrapyramidal signs and parkinsonism, which made the clinical diagnosis difficult [15,16].

Systematic genetic investigation of the available exome sequencing data from the two siblings (IV:16 and IV:18) identified a previously reported homozygous regulatory variant, c.-70G>A, in the *TH* gene [5,6,7]. *TH* encodes tyrosine hydroxylase, a rate-limiting enzyme in catecholamine biosynthesis that converts L-tyrosine to L-DOPA, the precursor of dopamine [17]. Although the exact global prevalence remains unknown, TH deficiency is estimated to affect 1 to 9 per million individuals in the European population (Orphanet, ORPHA:101150) and is characterized by considerable phenotypic variability [18]. Previously, TH deficiency was categorized into two major types: type A, characterized by an infantile onset, progressive hypokinetic rigid syndrome, and dystonia, with a generally favorable response to L-DOPA, and type B, characterized by neonatal onset with complex encephalopathy and a less favorable response to L-DOPA [19]. However, now considerable clinical variability has been reported among affected individuals, indicating that TH deficiency can cause a broader phenotypic spectrum than these two classical categories alone, involving parkinsonism, intellectual disability, ptosis, oculogyric crisis, tremors, severe hypokinesia, limb hypertonia, hyperreflexia, diurnal fluctuation of symptoms and paroxysmal periods of lethargy [20,21,22,23,24]. Although most disease-causing variants have been identified in coding or splice-site regions of *TH*, pathogenic regulatory variants affecting TH expression have also been reported [22,23,25,26,27].

TH deficiency causes low endogenous L-DOPA production that impacts dopamine production in the brain [19]. Administration of levodopa bypasses this impaired step by directly supplying L-DOPA, while carbidopa reduces its peripheral metabolism, allowing more levodopa to reach the central nervous system. The resulting increase in dopamine availability improves nigrostriatal and basal ganglia motor signaling, thereby facilitating movement initiation and motor output (Figure 2) [28]. The restoration of nighttime motor function in all three siblings, therefore, provides additional physiological support for the molecular finding.

The c.-70G>A variant lies within the octameric cAMP response element (CRE) of the *TH* regulatory region, which is important for *TH* transcription. Functional studies of this region have shown that disruption of this CRE region reduces basal and cAMP-induced promoter activity. DNase I foot-printing further showed that changes within the CRE affected protein–DNA interactions, indicating that an intact CRE is important for normal *TH* transcription [29].

Comparison of affected individuals of the PKSOK family with previously reported individuals carrying the c.-70G>A variant shows phenotypic variability [5,6,7]. A six-month-old female infant with severe hypokinesia, rigidity, tremor, and oculogyric crises showed normalization of symptoms following L-DOPA administration [6], while another eighteen-month-old female patient carrying the c.-70G>A variant presented with motor delay, mild spasticity, toe walking, and episodic eye supraversion, showing a remarkable response to L-DOPA-carbidopa [5]. Discontinuation of treatment in the latter patient resulted in bradykinesia, hypomimia, and ptosis, whereas reintroduction of low-dose L-DOPA-carbidopa again produced an excellent clinical response. Thus, the marked improvement observed in the PKSOK siblings is consistent with the established L-DOPA responsiveness of *TH*-related disease (see Table 2).

Despite similarities in treatment response with previously reported cases of *TH* deficiency, the temporal pattern observed in family PKSOK remains distinctive. The affected siblings share early onset, developmental delay, hypokinetic features, and levodopa responsiveness with the broader spectrum of *TH*-related disease, but they do not show the persistent dystonia, severe rigidity, tremor, oculogyric crises, spasticity, or other prominent movement abnormalities described in several previously reported individuals [19,21,22,23,26]. Although diurnal fluctuation has been described in *TH* deficiency more broadly [30], such a pattern has not been reported among previously described individuals carrying the c.-70G>A variant summarized in Table 2 [5,6,7]. Thus, the pronounced nocturnal predominance observed in family PKSOK represents an unusual phenotypic manifestation associated with this regulatory variant and further expands the clinical spectrum of *TH* deficiency.

## 4. Conclusions

We report here three siblings with TH deficiency characterized by pronounced nocturnal akinesia, with relative preservation of daytime motor function. Identification and segregation of the homozygous regulatory *TH* c.-70G>A variant, together with the marked response to levodopa–carbidopa, support the molecular diagnosis. Although diurnal fluctuation is recognized within the broader spectrum of *TH* deficiency, the prominent nocturnal akinesia observed in this family has not been reported among previously described c.-70G>A cases, highlighting the phenotypic variability associated with this regulatory variant. Hence, these findings further emphasize the importance of considering TH deficiency in patients with nocturnal akinesia that is responsive to levodopa–carbidopa.

## Figures and Tables

**Table 1:** Nocturnal akinesia characteristics, neurological examination, and response to levodopa–carbidopa in the three affected siblings.

Individual	IV:16		IV:18		IV:21	
**Current age in years (at evaluation)**	21		17		9	
**Age of onset (years)**	≤1		≤1		≤1	
**Age of full phenotype prevalence (years)**	4		4.5		4.2	
**Gender**	Male		Male		Male	
**Intellectual disability**	Mild		Mild		Mild	
**Speech delay**	Yes		Yes		Yes	
**Psychomotor delay**	Yes		Yes		Yes	
**Behavioral problems**	Yes		Yes		Yes	
**Time of occurrence**	Sunset		Sunset		Sunset	
**Frequency**	Daily		Daily		Daily	
**Severity (mild, moderate, severe)**	Severe		Severe		Severe	
**Approximate duration of episode (hours)**	~10		~11		~10.5	
**Dose of levodopa/carbidopa given**	100 mg/25 mg		100 mg/25 mg		100 mg/25 mg	
**Neurological observations for symptoms during episode**	**Before treatment**	**After** **treatment**	**Before** **treatment**	**After** **treatment**	**Before** **treatment**	**After** **treatment**
**Alertness**	Preserved	Preserved	Preserved	Preserved	Preserved	Preserved
**Cranial nerves**	Partial impairment	Normal	Partial impairment	Normal	Partial impairment	Normal
**Motor strength (MRC grade)**	2	4	2	5	2	5
**Muscle tone**	Hypotonia	Normal	Hypotonia	Normal	Hypotonia	Normal
**Reflexes (0–4)**	2+	2+	2+	2+	2+	2+
**Plantar responses**	Flexor bilaterally	Flexor bilaterally	Flexor bilaterally	Flexor bilaterally	Flexor bilaterally	Flexor bilaterally
**Sensory examination (pain sensation and touch)**	Preserved	Preserved	Preserved	Preserved	Preserved	Preserved
**Coordination (finger-to-nose and heel-to-shin test)**	Unable to assess due to severe motor impairment	Normal	Unable to assess due to severe motor impairment	Normal	Unable to assess due to severe motor impairment	Normal
**Balance**	Unable to assess due to severe motor impairment	Normal	Unable to assess due to severe motor impairment	Normal	Unable to assess due to severe motor impairment	Normal
**Gait**	Unable to assess due to severe motor impairment	Normal	Unable to assess due to severe motor impairment	Normal	Unable to assess due to severe motor impairment	Normal
**Abnormal movements (tremors, dystonia, chorea etc.)**	Not observed	Not observed	Not observed	Not observed	Not observed	Not observed
**Speech**	Impacted	Improved	Impacted	Improved	Impacted	Did not improve initially, but with growing age, it is becoming better
**Treatment-related adverse effects**	None		None		None	

**Table 2:** Comparison of reported *TH* c.-70G>A cases.

Study	Ethnicity	Zygosity	Gender	Major Clinical Phenotype	Response to L-DOPA
Family PKSOK	Pakistani	Homozygous	Three males	Nocturnal hypotonia/akinesia, normal daytime motor function, delayed motor milestones, psychomotor delay, mild intellectual disability, and speech impairment.	Marked improvement of nocturnal motor symptoms Dose: 100 mg Levodopa/25 mg Carbidopa Duration: after every 24 h
Verbeek et al., 2007 [7]	Dutch	Homozygous	Four individuals ^#^	TH deficiency, reduced CSF HVA concentrations with normal 5-HIAA, variable age of onset among affected individuals.	Not reported
Ormazabal et al., 2011 [5]	NA	Compound heterozygous *	Female	Motor delay, mild spasticity/toe walking, episodic eye supraversion; after treatment withdrawal: bradykinesia, hypomimia, and ptosis.	Excellent response to low dose Dose: 1.5 mg/kg) Duration: after every 12 h
Ortez et al., 2015 [6]	Spanish	Compound heterozygous *	Female	Onset at 6 months, oculogyric crisis, severe tremor, severe hypokinesia, rigidity, normal cognition, Type A TH deficiency.	Normalization of symptoms Dose and duration not mentioned

* c.-70G>A and c.1197+25_1391del; ^#^ 4 individuals from two unrelated families gender not specified; CSF: Cerebrospinal fluid, HVA: Homovanillic acid, 5-HIAA: 5-hydroxyindoleacetic acid.

## Data Availability

The original contributions presented in this study are included in the article/Supplementary Material. Sequencing data is not publicly available due to individual confidentiality and privacy restrictions but may be made available by the corresponding author upon reasonable request, subject to applicable ethical and confidentiality considerations. Further inquiries can be directed to the corresponding author(s).

## Supplementary Materials

The following supporting information can be downloaded at https://www.mdpi.com/article/10.3390/ijms27209068/s1.
