# Supplementary material for: Three Cases of Levodopa–Carbidopa Responsive Nocturnal Akinesis in Male Children of an Inbred Family in Rural Baluchistan, Pakistan
Source: Int J Mol Sci. 2026 Oct 13;27(20):9068. doi: 10.3390/ijms27209068 (PMC13704894; doi:10.3390/ijms27209068)
Supplement: Supplementary file 1 [file ijms-27-09068-s001.zip › Supplementary Table S1.pdf]

Supplementary Table S1. Hematological and metabolic laboratory findings of affected individuals

Test	Unit	Normal Range for testing lab	IV:16	IV:18	IV:21
**Hb**	g/dL	13-18	14.6	15.6	15.1
**RBC**	$\times 10^{12}$/L	4.5-6.5	4.8	4.9	5.2
**HCT**	%	38-52	44	46	45
**MCV**	fL	75-95	85	89	91
**MCH**	pg	26-32	29	30	28
**MCHC**	g/dL	30-35	33	32	33
**Platelets**	$\times 10^{9}$/L	150-400	298	300	315
**WBC**	$\times 10^{9}$/L	4-11	7.9	6.8	8.5
**Neutrophils**	%	40-75	64	60	58
**Lymphocytes**	%	20-50	32	36	36
**Monocytes**	%	02-10	2	2	4
**Eosinophils**	%	01-06	2	2	2
**Lactate**	mg/dL	5-20	12	16	9
**Ammonia**	μmol/L	16-60	24	31	48
